# Determination of oral feeding skills in late preterm, early term, and full-term infants using the neonatal oral feeding monitor (NeoSAFE)

**DOI:** 10.1186/s13052-025-01867-2

**Published:** 2025-02-07

**Authors:** Ayse Ecevit, Balkar Erdogan, Deniz Anuk Ince, Meltem Aksu, Sezin Unal, Özden Turan, Ahmet Saracoglu, Aylin Tarcan

**Affiliations:** 1 Faculty of Medicine, Department of Pediatrics, Division of Neonatology, Baskent University, Ankara, Turkey; 2 Middle East Technical University, Technology Transfer Office (TTO) KuartisMED Medical Company, Ankara, Turkey

**Keywords:** Oral feeding skills, Deglutition, Deglutition disorders, Newborn infants, Preterm, Newborn feeding assessment, Swallow detection, Swallow respiration coordination assessment, NeoSAFE

## Abstract

**Background:**

Mature oral feeding is a complex function involving numerous muscles and nerves, typically developing between the postmenstrual age of 34–36 weeks in newborn infants. The objective of this study was to analyze the oral feeding skills of healthy late preterm, early term, and full-term infants using a neonatal oral feeding monitor.

**Methods:**

We used the oral feeding parameters reported by NeoSAFE which is a certified medical device, to assess the swallowing and swallow-respiration coordination in newborn infants. Oral feeding parameters were recorded over a 2-minutes long bottle-feeding session. The total swallow count, swallow time, maximum rhythmic swallows, resting interval duration, time between rhythmic swallows and inspiration after swallow count were recorded by NeoSAFE. We planned to examine the relationship of oral feeding parameters according to the gestational age. We also investigated whether the coordination of swallowing and respiration changes with respect to gestational age in newborn infants.

**Results:**

A total of 88 infants were included; 34 late preterm, 34 early term, and 20 full term. The gestational age was found to have significant negative correlation with the average time between rhythmic swallows and positive correlation with the swallow time. Feeding volume was found to have a negative correlation with the resting interval duration and average time between rhythmic swallows. It was also found that the feeding volume has a positive correlation with total swallow count, swallow time, maximum rhythmic swallow and inspirium after swallow count.

**Conclusion:**

Although the oral feeding skills of infants at 34 weeks gestation are still developing, this study identified differences in oral feeding skills among late preterm, early term, and full-term infants when assessed using a neonatal swallow and respiration detection system. However, conducting larger cohort studies using NeoSAFE would be beneficial for guiding oral feeding approaches in infants.

**Trial Registration:**

Not applicable.

## Background

Efficient and safe oral feeding as well as optimal growth, rely on the acquisition of mature oral feeding skills [1–3]. Effective and safe oral feeding is one of the criteria for the hospital discharge of preterm infants as outlined by the American Academy of Pediatrics [4]. Even late-preterm and early-term infants are at risk of delayed attainment of effective oral feeding skills when compared to full-term infants [5]. This issue leads to prolonged hospital stays, increased readmission to the hospital, maternal stress, decreased mother-infant interaction, decreased breast milk supply, indirect hyperbilirubinemia, hypoglycemia, and dehydration [6–9].

Development of oral feeding skills in newborn infants is a complex process, with immature oro-motor development and effective coordination of sucking, swallowing and respiration being the critical factors [10–17]. Sucking performances are regulated by an organized neuronal network consisting of a suck Central Pattern Generator [18]. This neuronal network is responsible for generating the rhythmic patterns of muscle contractions required for sucking which are essential for both non-nutritive and nutritive sucking activities. This network also plays an important role in oral feeding. Oral feeding skills gradually develop in preterm infants and are often completed between 34 and 36 weeks PMA [19]. Development of oral feeding skills, particularly in coordination of sucking, swallowing and respiration is crucial in preventing aspiration in newborn infants [20–21]. The schedule for hospital discharge is usually extended for very preterm infants due to the delays in this development process [22].

Evaluating the newborn oral feeding, particularly the swallowing phases, still lacks an objective and non-invasive method for recognition. Methods such as video fluoroscopic swallowing study, manometry, and fiberoptic endoscopy are often used, but they involve costly and invasive procedures [23, 24]. Furthermore, video fluoroscopic swallowing study exposes infants to radiation. On the other hand, İnce et al. demonstrated the sequential swallowing-resting events using an acoustic method, measuring their durations by a digital stethoscope [25]. That study revealed that preterm infants reached the maturation of the swallowing level of the term infants at 34 weeks PMA at the earliest in preterm infants. Based on our previous study, NeoSAFE, a neonatal oral feeding monitoring device was developed. This is the first oral feeding monitor for newborn infants, to record and detect the swallowing sounds, and reporting their relationship with instantaneous respiratory data using machine-learning methods [26].

In the current study, our objective was to determine the oral feeding skills of healthy late preterm, early term, and full-term infants who were bottle-fed [27]. We used the oral feeding parameters reported by NeoSAFE to evaluate the swallowing patterns and development of swallow-respiration coordination of these newborns. Furthermore, our goal was to explore the differences in oral feeding parameters between late preterm and early term infants as compared to full-term infants.

## Methods

### Study design

The study was conducted during examinations in neonatal outpatient clinic at the Baskent University Hospital. We included otherwise healthy late preterm, early term, and full-term infants who were 3–10 days old and with informed parental consent. This study excluded infants with medical complications such as multiple congenital abnormalities, genetic syndromes, current sepsis, other infections and major neurologic complications.

**Fig. 1:**
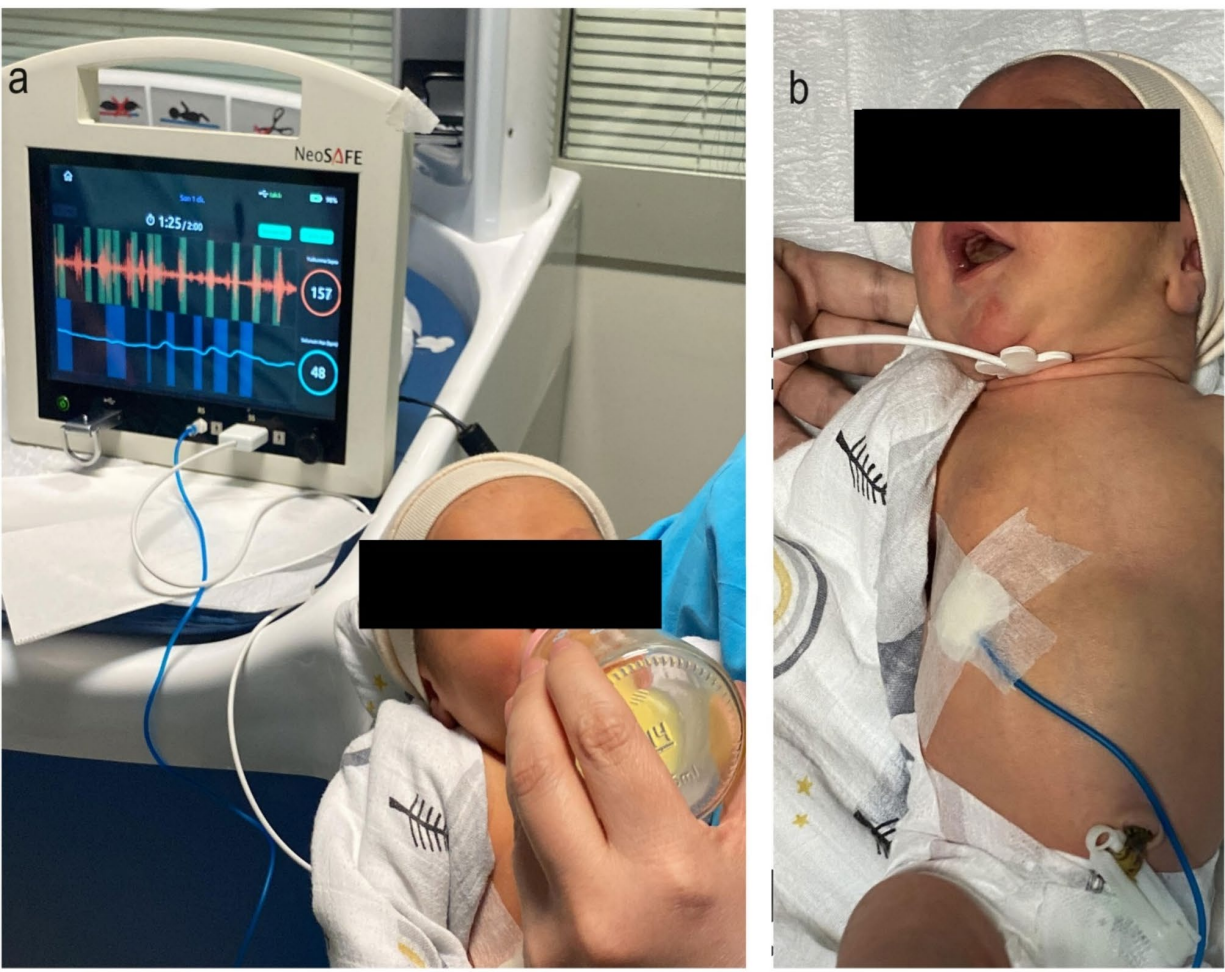
**a.** Recording was taken with infant positioned at a 30-degree supine angle in the mother’s lap. The swallow and respiration traces are shown on the display indicating the instances of swallows (upper trace) and inspirations (lower trace). **b**. The sites of the probes on the newborn. The swallow probe is situated under the chin and the respiration probe is attached on the subject’s right abdomen

We used NeoSAFE to evaluate the oral feeding skills of the newborns. For each subject, NeoSAFE measured the total swallow count, swallow time, maximum rhythmic swallows, resting interval duration, average time between rhythmic swallows, inspiration after swallow count, duration of inspirium after swallow and average respiration rate (Table 1) obtained during 2 min of bottle-feeding sessions. For each newborn, an unused bottle and a rubber bottle nipple were used for the feeding session. It was ensured that these feeding sessions were performed on each subject at least 2 h after the last feed. During recording, infants were positioned at a 30-degree supine angle on their mothers’ laps as seen in Fig. 1a. The recordings were obtained in a quite environment where the average sound level in the room did not exceed 45dB.

**Table 1:** Description of NeoSAFE feeding parameters evaluated in this study

Parameter	Description
Total Swallow Count	The number of swallows (both rhythmic and non-rhythmic) detected throughout the feeding session
Swallow Time	Average duration of individual swallows in seconds
Resting Interval Duration	Total duration of resting intervals (in seconds) when rhythmic swallowing is not observed
Maximum Rhythmic Swallow	Maximum number of consecutive swallows among the rhythmic swallows
Average Time Between Rhythmic Swallows	Average time between two (consecutive) rhythmic swallows in seconds
Inspirium After Swallow Count	Number of inspirium after swallow events detected during feeding session
Duration of Inspirium After Swallow	Average duration (in seconds) of detected inspirium after swallow events
Respiration Rate	Average respiration rate measured during feeding in breaths per minute

Beside the parameters reported by NeoSAFE, we recorded the gestational age, birth weight, current weight, postnatal day and volume of the feeding for each recording. These data were logged and statistically inspected according to the categories presented in the results section.

### Description of NeoSAFE

**Fig. 2:**
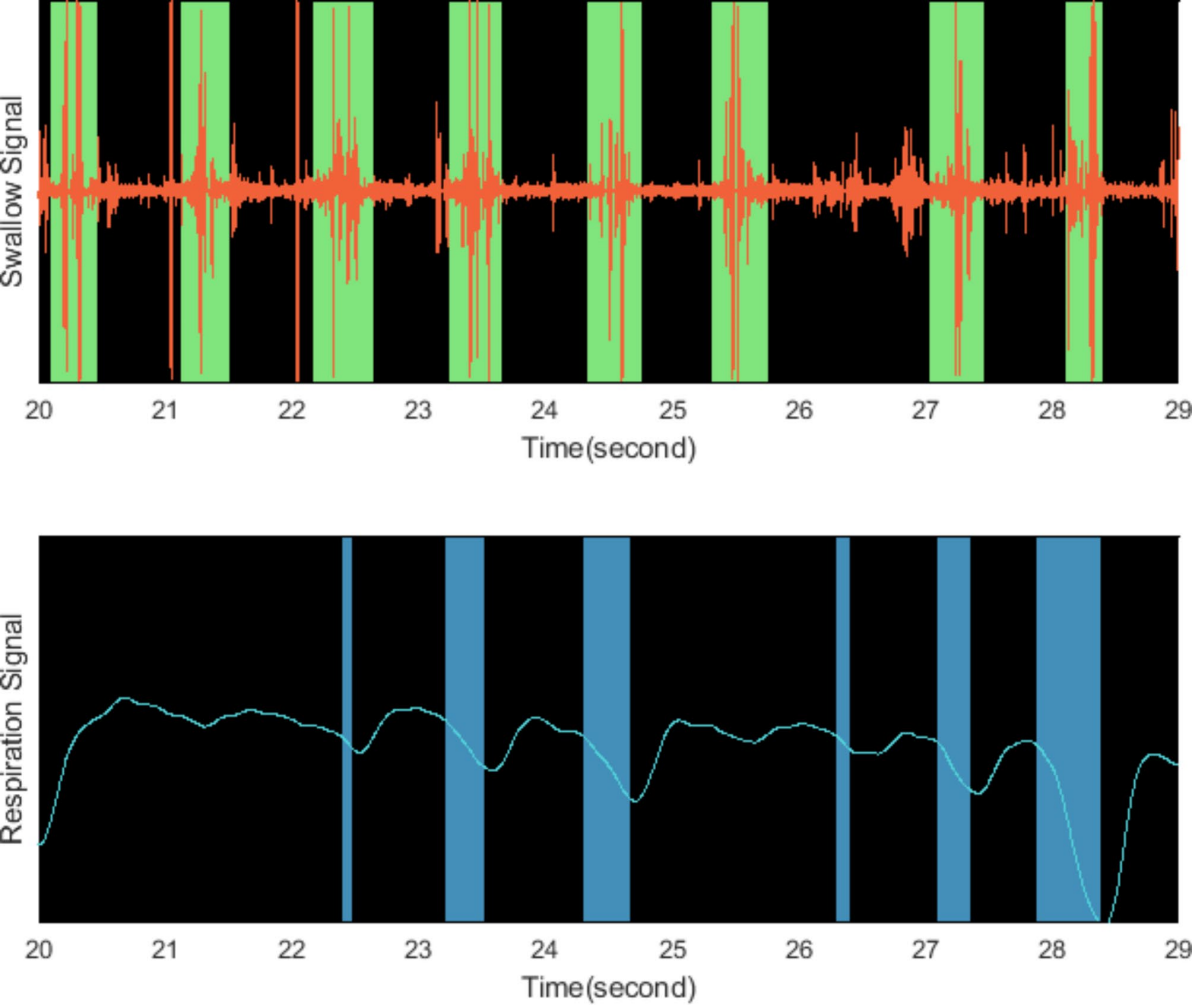
Swallow sound (top) and respiration (bottom) signals displayed on NeoSAFE oral feeding monitor during the feeding session. The swallow instants are marked by green rectangles on the sound signal and the inspirium instants are marked by blue rectangles on the respiration trace

The data analyzed in this study is acquired using NeoSAFE which is a newborn oral feeding monitor that measures the swallows and respiration signals of the newborn and provides oral feeding parameters related to the feeding session. NeoSAFE (KM1001-PM-A1) is a Class IIa CE-certified medical device developed in accordance with the 93/42/EEC. It aims for a practical, standardized and data driven assessment and monitoring method to evaluate newborn oral feeding skills. The device is operated using two measurement probes: (1) a swallow sensor, placed under the chin and (2) a respiration sensor placed over the abdomen region of the baby (Fig. 1.b). The swallow sensor is simply an acoustic transducer that collects the sound signals emanating from the baby’s oral cavity and epiglottis. The device identifies the swallow sounds from the raw data using acoustic signal processing and machine learning methods. On the other hand, the respiration sensor is a pneumatic type transducer in which the millimetric expansion and contraction of the sensor are measured and sensed by a pressure gauge located inside the device. The inhale and exhale activity can be detected by this method as the cyclic movement of the abdomen during the respiration alters the pressure applied on the sensor. Once the sensors are placed on the subject and the measurement is started, a 2-minutes long feeding session is recorded by the device. During the recording, the detected swallow events are marked on the swallow trace, and inspiration instants are indicated on the respiration trace on the software. Figure 2 shows marked swallow and inhalation instants during the swallow and respiration signals displayed on NeoSAFE. The acoustic signal captured by the swallow probe is provided as a sound waveform and the swallow instants are marked by green rectangles on the swallow trace. Meanwhile, the respiration trace displays the variation of the pressure exerted on the respiration probe and the durations of the inhale instants are marked with blue rectangles. The swallow and respiration detection are performed instantly, during the recording session. At the end of the feeding session, the detected swallows and inhale/exhale activity are used to extract total swallow count, maximum rhythmic swallow count, minimum rhythmic swallow count, average time between rhythmic swallows, resting interval count, resting interval duration, inspirium after swallow count, inspirium after swallow duration and average respiration rate parameters. The device generates a report including the values of these parameters calculated on the entire recording. In addition to the report, the raw data (swallow and the respiration signals) of each feeding session were extracted to an external memory medium to be analysed by the users. Therefore, one can visualize, listen, and review each recording for further inspection. The values of these parameters are used in the statistical analysis performed for this study.

### Statistical analysis

The data were evaluated in the statistical package program IBM SPSS Statistics Standard Concurrent User V26 (IBM Corp., Armonk, New York, USA). Descriptive statistics were given as number of units (n), percentage (%) and mean ± standard deviation ($\:\stackrel{-}{\text{x}}$ ± sd). The normal distribution of the data of numerical variables was evaluated with the Shapiro-Wilk normality test. When the differences between groups were to be examined, One Way ANOVA test was used if the data met normal distribution conditions, and Kruskal Wallis H test was used otherwise. When multiple group comparison was statistically significant the Bonferonni correction was applied for pairwise comparisons and a *p*-value < 0.015 was considered statistically significant. When the relationships between numerical variables were examined, Spearman’s rank correlation coefficient was used since the data were not distributed normally. A value of *p* ≤ 0.05 was considered statistically significant. A priori power analysis was conducted to determine the minimum sample size required to test the significance of the difference in total swallow count among the three groups. Results indicated the required sample size to achieve 80% power for detecting a large effect, at a significance criterion of α = 0.05, was *n* = 66 [ANOVA: Fixed effect, omnibus, one-way].

## Results

A total of 90 parents gave informed consent for their newborns to be included in the study. During the study, two of the term infants were excluded due to parental wish. Analysis of 88 newborns were performed, 34 (38.6%) for the late preterm group, 34 (38.6%) for the early term group and 20 (22.7%) for the full-term group. Among these infants, the gestational age was 37.2 ± 1.6 weeks, the birth weight was 2987 ± 566 g, the current weight was 2928 ± 573 g. The feeding volume measured during the 2 min of feeding session was 17.6 ± 4.6 ml and was similar in three groups. The characteristics of the included infants are given in Table 2.

**Table 2:** The group characteristics of the infants

	Late Preterm (*n* = 34)	Early Term (*n* = 34)	Full Term (*n* = 20)		*p* value	
Gestational age (weeks)	35.4 ± 0.8	37.7 ± 0.5	39.3 ± 0.6		< 0.001*	
Birth weight (g)	2570 ± 430	3076 ± 424	3543 ± 427		< 0.001*	
Postnatal age (days)	4 ± 2	4 ± 3	4 ± 2		0.919	
Current weight (g)	2483 ± 445	3052 ± 417	3474 ± 405		< 0.001*	
Feeding volume (ml)	17.4 ± 4.3	17.5 ± 5.1	18.0 ± 5.5		0.636	

* Pair-wise comparisons show statistically significant differences between all groups

Values are presented as mean ± standard deviation

Table 3 shows the statistics of the feeding parameters reported by NeoSAFE for each infant group. The total swallow count, swallow time, maximum rhythmic swallows, inspirium after swallow count, and duration of inspirium after swallow were observed to be higher in infants born at greater gestational ages; however, these results were not statistically significant. Additionally, the resting interval duration and average time between rhythmic swallows were found to be lower in infants born at higher gestational ages, yet these results were also not statistically significant.

**Table 3:** Feeding parameters reported by NeoSAFE with respect to the infant group

	Late Preterm	Early Term	Full Term	*p* value
Total swallow count (n) *	31.9 ± 17.2	37.9 ± 18.8	39.5 ± 21.4	0.310
**S**wallow time (secs)*	0.36 ± 0.25	0.60 ± 0.79	0.59 ± 0.56	0.298
Resting interval duration (secs) **#**	49.05 ± 27.88	55.04 ± 26.20	44.48 ± 21.83	0.335
Maximum rhythmic swallows (n) *	16.5 ± 10.7	20.9 ± 18.2	19.1 ± 11.4	0.609
Average time between rhythmic swallows (secs) *	0.87 ± 0.42	0.78 ± 0.40	0.73 ± 0.33	0.281
Inspirium after swallow count (n) *	11.6 ± 9.5	20.0 ± 20.7	20.7 ± 17.5	0.137
Duration of inspirium after swallow (secs) *	0.44 ± 0.82	0.76 ± 1.32	0.95 ± 1.23	0.326
Respiration rate (n/min) *	54 ± 20	56 ± 25	57 ± 20	0.834

Values are presented as mean ± standard deviation

*Kruskal Wallis, #ANOVA

Table 4 shows the correlation between the reported parameters and group characteristics, including gestational age, postnatal day, birth weight, current weight and feeding volume. Gestational age was found to be positively correlated with swallow time and negatively correlated with the average time between rhythmic swallows, although the correlation coefficients were low. There was a statistically significant, yet weak positive correlation between feeding volume and swallow count, swallow time, maximum rhythmic swallows and conversely, a statistically weak but significant negative correlation was found between feeding volume and resting interval duration, average time between rhythmic swallows and the respiration rate.

**Table 4:** Correlation values of the extracted feeding parameters regarding the gestational age, postnatal age, birth weight, measured weight, and feeding volume in all subjects

		Gestational Age	Postnatal Age	Birth Weight	Current Weight	Feeding Volume
Total Swallow Count	rho	0.175	0.119	0.009	0.036	0.297
	p	0.102	0.269	0.931	0.738	0.005
Swallow Time	rho	0.214	0.027	0.146	0.134	0.291
	p	0.045	0.802	0.174	0.213	0.006
Resting Interval Duration	rho	-0.084	0.011	-0.056	-0.063	-0.317
	p	0.434	0.919	0.604	0.561	0.003
Maximum Rhythmic Swallow	rho	0.142	0.023	0.004	0.019	0.351
	p	0.187	0.833	0.971	0.858	0.001
Average Time Between Rhythmic Swallows	rho	-0.224	0.029	-0.149	-0.171	-0.262
	p	0.036	0.791	0.166	0.111	0.014
Inspirium After Swallow Count	rho	0.247	0.049	0.192	0.178	0.288
	p	0.020	0.653	0.073	0.097	0.006
Duration of Inspirium After Swallow	rho	0.156	-0.069	0.073	0.104	0.042
	p	0.146	0.521	0.500	0.337	0.700
Respiration Rate	rho	-0.005	0.049	0.097	0.097	-0.264
	p	0.960	0.650	0.367	0.367	0.013
Spearman correlation analysis						

## Discussion

In this study, we analyzed parameters related to oral feeding skills reported by NeoSAFE, recorded from 88 healthy bottle-fed infants born between 34^0/7^ and 40 ^6/7^ weeks of gestation. In our study, the correlation analysis revealed a statistically significant negative correlation between gestational age and the average time between rhythmic swallows. Also, gestational age positively correlates with the swallow time. As gestational age increases, the increase in swallow time along with the decrease in average time between rhythmic swallows, can be interpreted as the requirement of more frequent inspiriums in short periods following longer swallow times. This can be an indication of mature oral feeding which is usually seen in term infants.

In addition, we observed that feeding volume increases with a higher swallow count, longer swallow time, and more maximum rhythmic swallows. These findings can be interpreted as infants with more mature swallowing function are able to consume higher feeding volumes. The increase in inspirium after swallow as feeding volume increases could suggest either that these infants are older in gestational age, or that the higher feeding volume requires greater effort, leading to more frequent inspiriums and swallows. In future studies, evaluating the changes in inspirium after swallows over the entire feeding session with longer recordings could help shed light on this topic. Meanwhile, the decrease in resting interval duration and average time between rhythmic swallows as feeding volume increases aligns with clinical observations, indicating that shorter resting periods are associated with an increase in feeding volume. In other words, the infants with more mature oral feeding skills tend to have a higher feeding volume. Although these correlations are weak, the results are promising and suggest potential clinical benefit.

There are some discussions about oral feeding success in preterm infants [28]. Definition of oral feeding success varies in the literature which necessitates further clarification. One definition of oral feeding success involves whether a newborn infant can consume the full prescribed oral volume while considering its impact on physiological stability. Likewise, if newborn infants can orally consume 100% of prescribed volume within 20–30 min, it is also considered as a sign of oral feeding success [28]. Several methods exist for assessing the oral feeding maturity, analysis of sucking and swallowing behavior and oral feeding success in newborn infants, including ultrasound, nfant^®^ Feeding Solution, pressure transducers, NTrainer and Smart Bottle technology [29–33]. Ultrasound imaging is a non-invasive method for assessment of swallowing by visualizing the movement during oral feeding [32]. However, this approach requires a trained specialist to ensure accurate and objective results.

We previously examined feeding maturation by recording the swallowing sounds in preterm infants using a digital stethoscope during the follow-ups in the neonatal intensive care unit [25]. In this study, we demonstrated that by 34 weeks of PMA, preterm infants attained an oral performance level comparable to the 10th percentile value of term infants. Following the findings of our previous research, NeoSAFE was developed. Subsequently, we conducted a study employing this method, and the results were presented at the 55th ESPGHAN Meeting [26]. Upon examining the infants’ swallowing results with ultrasonography, it was noted that the data from NeoSAFE were similar to that of ultrasonography. However, the observational swallowing data was significantly higher than the swallowing data obtained through ultrasonography and NeoSAFE.

Insufficient maturation in the oropharyngeal phase of swallowing and a lack of coordination of sucking, swallowing and respiration can result in oropharyngeal aspiration, particularly in premature infants. There are various tools used for the diagnosis of oropharyngeal aspiration, including auscultation, radiography, fiberoptic endoscopic swallow evaluation, and video fluoroscopic swallowing study [23, 24]. However, these methods are invasive, costly, require specialized training, and may expose infants to radiation. Kamity et al. simultaneously employed video fluoroscopic swallowing study and fiberoptic endoscopic swallow evaluation for detecting penetration and aspiration in five preterm infants (PMA > 36 weeks) with dysphagia [34]. Another study that utilized video fluoroscopic swallowing study in observing swallows revealed the importance of this method in diagnosing oropharyngeal aspiration, emphasizing that appropriate referrals are crucial to prevent unnecessary radiation for preterm infants with risk for aspiration [35]. In our study, inspirium after swallow count was found to be weakly correlating with the feeding volume. In an ongoing study, we observe this relation to be more significant in term infants where the subjects exhibit higher swallow counts. Therefore, the term group can be allowed to have higher inspirium after swallow count and have less risk of aspiration due to their fairly well developed feeding skills. However, this is not the case for especially small preterm infants where an increase in the number of inspiration after swallow poses an aspiration risk. Nevertheless, NeoSAFE can be used to understand and recognize the risk of oropharyngeal aspiration in a non-invasive way, particularly in preterm infants.

Although the number of cases evaluated in our study is limited, we were able to demonstrate the potential use of NeoSAFE in exploiting the relation between gestational age and its swallow and respiration coordination related parameters. However, the small sample size may have increased the likelihood of Type II error, which could explain the lack of statistical differences between groups. Therefore, further studies with a larger number of cases are needed to establish normal values and create a reference range. Additionally, the limited duration of recording is a concern. Future studies should evaluate entire feeding sessions, as this may provide a different perspective on the characteristics of individual infants.

## Conclusion

It is well known that the oral feeding skills of infants at 34 weeks gestation are still developing, and this study identified slight differences in oral feeding skills when assessed using NeoSAFE among late preterm, early term, and full-term infants. We believe that investigating swallow count, maximum rhythmic swallow, inspirium after swallow count, resting interval duration and the average time between rhythmic swallows in relation to oral feeding skills will yield valuable insights for clinical decisions regarding safe discharge, ultimately benefiting newborn health. NeoSAFE has the advantages of being non-invasive, radiation free, data driven and easy to operate as compared to the conventional methods in oral feeding assessment. Conducting larger cohort studies using NeoSAFE would be beneficial for guiding oral feeding approaches in infants.

## Data Availability

Data cannot be shared openly but are available on request from authors:

## Abbreviations

PMA: Postmenstrual age
